# Male sexual ornament size is positively associated with reproductive morphology and enhanced fertility in the stalk-eyed fly *Teleopsis dalmanni*

**DOI:** 10.1186/1471-2148-8-236

**Published:** 2008-08-18

**Authors:** David W Rogers, Matthew Denniff, Tracey Chapman, Kevin Fowler, Andrew Pomiankowski

**Affiliations:** 1 The Galton Laboratory, Research Department of Genetics, Evolution and Environment, University College London, 4 Stephenson Way, London, NW1 2HE, UK; 2 Division of Cell and Molecular Biology, Imperial College London, Imperial College Road, London, SW7 2AZ, UK; 3 Department of Genetics, University of Leicester, University Road, Leicester, LE1 7RH, UK; 4 School of Biological Sciences, University of East Anglia, Norwich, NR4 7TJ, UK; 5 Collegium Budapest, Szentháromság utca 2, H-1014 Budapest, Hungary; 6 CoMPLEX, University College London, Gower Street, London, WC1E 6BT, UK

## Abstract

**Background:**

Exaggerated male ornaments and displays often evolve in species where males only provide females with ejaculates during reproduction. Although "good genes" arguments are typically invoked to explain this phenomenon, a simpler alternative is possible if variation in male reproductive quality (e.g. sperm number, ejaculate content, mating rate) is an important determinant of female reproductive success. The "phenotype-linked fertility hypothesis" states that female preference for male ornaments or displays has been selected to ensure higher levels of fertility and has driven the evolution of exaggerated male traits. Females of the stalk-eyed fly *Teleopsis dalmanni *must mate frequently to maintain high levels of fertility and prefer to mate with males exhibiting large eyespan, a condition-dependent sexual ornament. If eyespan indicates male reproductive quality, females could directly increase their reproductive success by mating with males with large eyespan. Here we investigate whether male eyespan indicates accessory gland and testis length, and then ask whether mating with large eyespan males affects female fertility.

**Results:**

Male eyespan was a better predictor of two key male reproductive traits – accessory gland and testis length – than was body size alone. This positive relationship held true over three levels of increasing environmental stress during the maturation of the adult accessory glands and testes. Furthermore, females housed with a large eyespan male exhibited higher levels of fertility than those with small eyespan males.

**Conclusion:**

Male eyespan in stalk-eyed flies is subject to strong directional mate preference and is a reliable indicator of male reproductive quality – both because males with larger eyespan have bigger accessory glands and testes, and also as they confer higher fertility on females. Fertility enhancement may have arisen because males with larger eyespan mated more often and/or because they transferred more sperm or other substances per ejaculate. The need to ensure high levels of fertility could thus have been an important selective force in the coevolution of female preference and male eyespan in stalk-eyed flies. Our results support the phenotype-linked fertility hypothesis and suggest that it might be of general importance in explaining the evolution of exaggerated male ornaments and displays in species where males only provide females with ejaculates during reproduction.

## Background

Female mate preferences can evolve for exaggerated male traits that are indicators of male quality [1]. In resource-free mating systems in which males only provide females with ejaculates, it is often assumed that choosy females benefit through indirect genetic benefits to their offspring [2]. However, ejaculates vary in the amount of accessory substances as well as the number and quality of sperm transferred, and these constituents are important determinants of female fertility, fecundity and longevity [3]. Consequently, if male ornamentation also signals ejaculate quality, this is likely to be an additional force favouring the evolution of female mate preferences.

In this paper, we investigate whether male ornamentation signals reproductive quality in the stalk-eyed fly *Teleopsis dalmanni *(synonym *Cyrtodiopsis dalmanni *[4]), in particular, the ability of males to confer fertility. There is evidence that variation in fertility is an important component of female fitness in *T. dalmanni*. Multiple copulations are required by females to achieve and maintain high fertility [5]. This is probably because of the small size of the male spermatophore [6,7] and the small number of sperm stored following a single copulation (approximately 65 [8]). This effect is compounded by X-linked meiotic drive, which is found in 13–17% of field collected males [9]. Meiotic drive impairs the development of Y-bearing sperm, causing lowered fertility in females mated to drive males [10,11]. Consistent with these findings, less than 40% of eggs laid by field-caught females failed to hatch, though the exact cause of this has yet to be established [12].

Male reproductive quality is associated with the size of the accessory glands and testes. These internal reproductive organs are very small at eclosion, and then dramatically increase in size, allowing males to attain sexual maturity three to four weeks later [13]. Accessory gland growth rate is positively correlated with the time required to reach sexual maturity [13] while the size of the mature accessory glands is both phenotypically [13,14] and genetically [15] correlated with male mating frequency. The ability of a male to mate multiply is likely to be an important contributor to female fertility, as matings are concentrated in the periods shortly before dusk and after dawn when single males mate with several females [16]. Testis size is positively correlated with the number of sperm stored in a female's spermathecae following copulation [17] and thus is also likely to affect female fertility.

A number of experiments have established that females prefer to roost and copulate with males with larger eyespan [18-20]. Hence it is possible that male eyespan acts as a signal of male reproductive quality and so leads to elevated fertility in choosy females. Note however that while eyespan is a highly condition-dependent trait which is sensitive to environmental stress during larval development [21-23], external morphology is fixed at eclosion [24]. This seems likely to limit the effectiveness of eyespan as a signal of accessory gland and testis size as these traits are strongly influenced by environmental stress during their maturation in adult flies [13]. Consequently there is a compelling need to explicitly investigate whether male eyespan predicts the growth and size at maturity of the internal reproductive organs, and whether it influences female fertility.

The possibility that fertility assurance is a major selective force in the evolution of female mate preferences for exaggerated male sexual ornaments has been discussed most extensively under the heading of the "phenotype-linked fertility hypothesis". This hypothesis originally linked fertility assurance to extra-pair matings in socially monogamous birds [25,26] but has since been generalized to all cases where sexual ornaments covary with male fertility traits [27,28]. More recently, the idea has been framed within the handicap principle, with the implication that the covariance between male ornaments and fertilizing efficiency arises because both are condition-dependent traits [29]. So males in good condition not only develop more attractive ornaments but also make greater investment in male fertility traits.

Empirical evidence for the hypothesis is ambiguous [27,30]. For example, studies using commercial strains or commercial/wild hybrids of domestic fowl have largely supported the hypothesis [29,31], whereas no relationship between male comb size and testis size was found in a captive population of Red Jungle fowl, the conspecific ancestor of domestic fowl [32]. The strongest support for the phenotype-linked fertility hypothesis comes from Wagner & Harper's [33] study of field crickets. Male chirp rate is positively correlated with the number of sperm transferred and females mated to males with higher chirp rates exhibited higher lifetime fertility. However, Wagner & Harper [33] may have exaggerated the importance of this finding as females were restricted to a single mating; given the opportunity, female field crickets normally remate at high frequency [34]. Furthermore, positive results were only observed in females fed a restricted diet; females fed a standard diet received no fertility benefits from males with high chirp rates.

In this paper we consider three fundamental requirements for the phenotype-linked fertility hypothesis to be supported. First, the male ornament or display must be subject to female mate preference. Second, attractiveness of the male ornament or display must be positively correlated with appropriate measures of male reproductive quality. This relationship needs to be made across the range of environments encountered in natural populations, as associations between fitness components can disappear or reverse direction in different environments [35,36], so single environment measures could be misleading. Third, females that mate with males possessing exaggerated ornaments or displays must exhibit higher fertility than females that mate with males with lower trait values. Otherwise positive correlations between ornament expression and male reproductive traits are not likely to be relevant to the issue of adaptive female preference [37].

In *T. dalmanni*, directional female preference for large eyespan males has already been documented [17-19], so we did not investigate it further here. We examined whether male eyespan was positively correlated with the growth of the accessory glands and testes as a way of gauging variation in male reproductive quality. These relationships were measured under a range of nutritional regimes, which cause environmental stress during adult development. We then tested whether females mated to large eyespan males exhibited higher fertility than females mated to small eyespan males.

## Results

### Male eyespan and reproductive organ size

Males used in this study were raised under high larval nutritional stress and exhibited high variation in thorax length (CV = 9.15, range = 1.65 – 2.72 mm) and eyespan (CV = 15.35, range = 4.25 – 8.73). Variation was slightly lower than levels observed in the natural populations from which the stock population was founded (thorax length CV = 16.51, range 1.46 – 3.11 mm; eyespan CV = 23.57, range 3.88 – 10.61 mm, N = 66, S. Cotton & A. Pomiankowski, unpublished data) which may reflect altered conditions or selection for adaptation to the laboratory setting. Reproductive organ size was strongly positively allometric. This variance was accounted for by including thorax length in initial models of accessory gland length (*F*_1,377 _= 94.22, *p *< 0.001) and testis length (*F*_1,378 _= 99.77, *p *< 0.001). Variance associated with differences in male age was removed by including age in initial models of accessory gland (*F*_1,377 _= 345.54, *p *< 0.001) and testis length (*F*_1,378 _= 38.18, *p *< 0.001).

Variation in adult nutritional stress (0% corn, 25% corn, and 75% corn diets) caused marked differences in reproductive organ size. As expected, males showed reduced accessory gland (*F*_2,377 _= 84.06, *p *< 0.001) and testis length (*F*_2,378 _= 19.82, *p *< 0.001) when subject to higher levels of nutritional stress during adult development. Multiple pairwise comparisons (Tukey HSD tests) revealed significant differences in accessory gland lengths between all three diets in accessory gland length (75% corn > 25% corn > 0% corn). Similarly, testis length of males reared on 0% corn were significantly smaller than those raised on 75% corn (testis length of males raised on 25% corn were not significantly different from those of males raised on either other diet). Both internal reproductive organs also exhibited slower rates of growth under high levels of adult nutritional stress (diet × age interaction: accessory glands *F*_2,377 _= 15.51, *p *< 0.001; testes *F*_2,378 _= 7.69, *p *< 0.001).

In order to investigate whether eyespan predicted reproductive organ size, we chose an experimental design in which flies were sorted into large and small residual eyespan categories, across a large range of male eyespan and body size (see Methods). This controlled for allometric scaling between body size and eyespan [23], as the two classes of males differed in eyespan (mean ± s.e.: large = 6.94 ± 0.06 mm, small = 6.46 ± 0.08 mm; *t*_425 _= 4.97, *p *< 0.001) but not thorax length (large = 2.17 ± 0.01 mm, small = 2.18 ± 0.02 mm; *t*_425 _= 0.76, *p *= 0.449).

**Figure 1:**
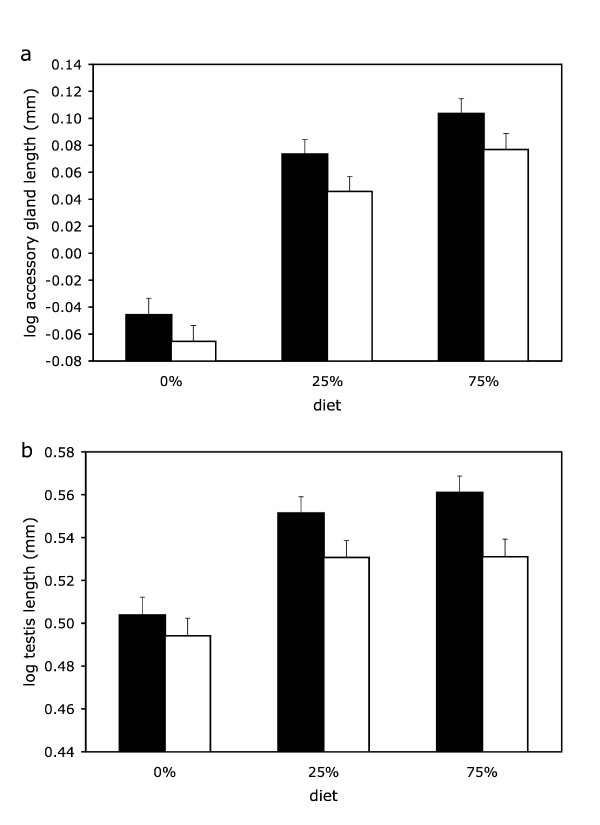
**The effect of residual male eyespan and adult nutritional stress on growth of (a) the accessory glands and (b) the testes**. Data represent least squares means ± s.e. log reproductive organ length (at average values of thorax length and age) for large residual eyespan (filled bars) and small residual eyespan (open bars) males across the three diets.

Adding eyespan (as a categorical variable) to the initial models described above explained a significantly larger proportion of the variance in both accessory gland (*F*_1,376 _= 6.98, *p *= 0.009, Fig 1a) and testis length (*F*_1,377 _= 9.67, *p *= 0.002, Fig 1b) than the initial models did. That is, eyespan was a better predictor of reproductive organ size than body size alone: males with large residual eyespan had bigger accessory glands and testes than did males with small residual eyespan. Adding absolute values of eyespan (instead of eyespan category) to the initial models produced qualitatively similar results; absolute eyespan significantly improved the fit of the models of accessory gland (F_1,376_ = 14.02, *p *< 0.001) and testis length (F_1,377_ = 26.53, *p *< 0.001). Estimating the regression coefficient (*b *± s.e.) of reproductive organ size on absolute eyespan indicated that males gained 0.123 ± 0.013 mm in accessory gland length and 0.291 ± 0.025 mm in testis length per mm increase in absolute eyespan (eyespan range: 3.96 – 9.21 mm) – equivalent to roughly 11% and 8% increase respectively per mm increase in absolute male eyespan.

Finally, we found no evidence that the reproductive organs of large residual eyespan males responded differently to nutritional stress than those of small residual eyespan males (eyespan × diet interaction, accessory glands: *F*_2,374 _= 0.061, *p *= 0.94; testes: *F*_2,375 _= 0.78, *p *= 0.4611). Indeed, the difference in accessory gland and testis length between large and small eyespan males was remarkably constant across stress levels (Fig. 1).

### Male eyespan and fertility

**Figure 2:**
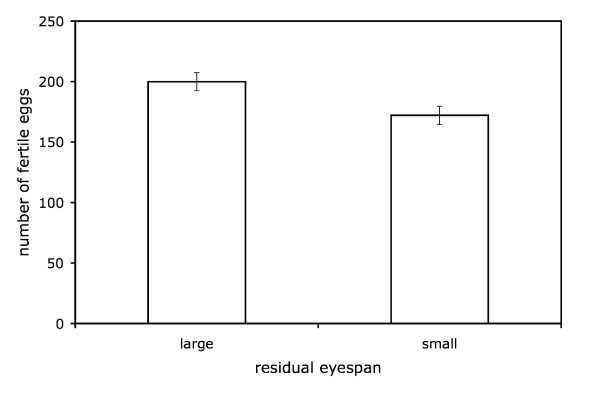
**The effect of male eyespan on fertility**. Least squares means ± s.e. number of fertile eggs laid in 32-day period following mating (at average levels of fecundity) by large residual eyespan and small residual eyespan males.

The number of fertile eggs laid by groups of 8 females mated to a single male for 24 hours was assessed over the subsequent 32 days. Both female fecundity (F_1,71_ = 107.21, *p *< 0.001) and male thorax length (F_1,71_ = 15.96, *p *= 0.002) predicted the number of fertile eggs laid. Adding male eyespan (as a categorical variable) to this model significantly improved the fit (*F*_1,71 _= 6.80, *p *= 0.011, Fig. 2). Females mated to large residual eyespan males laid significantly more fertile eggs than did females mated to small residual eyespan males (large = 199.83 ± 7.44, small = 172.07 ± 7.54, least squares mean ± s.e.). Thus, male eyespan predicted significantly more variance in fertility than body size alone. Adding absolute values of male eyespan (instead of eyespan category) to the basic model of body size and fecundity produced qualitatively similar results; eyespan significantly improved the fit of the model (*F*_1,71 _= 7.95, *p *= 0.006). Estimating the regression coefficient (*b *± s.e.) of the number of fertile eggs on eyespan indicated that females laid 15.38 ± 4.25 more fertile eggs per mm increase in male absolute eyespan (eyespan range: 3.96 – 9.21 mm) – equivalent to roughly an 8% increase in female fertility per mm increase in male absolute eyespan.

## Discussion

Why do females prefer elaborate male ornaments and displays in species where females receive only ejaculates from males? Under the pervasive assumption that "fertility is seldom likely to be limited by sperm production" [38], indirect genetic benefits are typically evoked. This has resulted in the relative neglect of an obvious alternative, that females choose ornamented males to ensure they receive sufficient sperm or other components of the ejaculate to fertilise their eggs.

In *T. dalmanni*, eyespan and all other external morphological traits are permanently fixed in size shortly after eclosion [24] and cannot change in response to the adult environment. In contrast, the accessory glands and testes only start to develop after eclosion. In this paper, we established a range of adult nutritional stresses which affected the size and growth rate of the male accessory glands and testes. We then investigated variation in male eyespan (after controlling for body size), the principal trait subject to female mate preference [18-20]. Despite this mismatch in developmental periods, male eyespan was a strong predictor of internal reproductive organ size. How can this association be explained?

Previous work on *T. dalmanni *has demonstrated that male eyespan is highly sensitive to stress during the larval stage [21,23], and certain genotypes are capable of producing large eyespan across a range of larval environments [39]. In more stressful environments, variation in eyespan is greater [22] and causes an amplification of genotypic differences between males [39]. These results are consistent with the handicap principle, which assumes that high quality males pay lower marginal costs for the production of larger eyespan than do low quality males [40].

Could male internal reproductive organ size be subject to similar condition-dependent costs? The handicap principle predicts that under harsh (0% corn) adult conditions, high quality males should still be able to develop large accessory glands and testes, whereas low quality males will be unable to do so (because of the higher marginal cost of reproductive organ size development felt by low quality males). But under benign adult conditions, all males will get access to sufficient resources independent of their quality. So differences between low and high quality males in the size of accessory glands and testes should be reduced under benign (75% corn) adult conditions (because the marginal cost of reproductive organ size development is much more similar in low and high quality males). Assuming that male eyespan is an indicator of quality, the correlation between eyespan and internal reproductive ornament size should decline as adult conditions become less stressful. Our results do not support this prediction. Large eyespan males consistently developed larger accessory glands and testes than small eyespan males under high (0% corn), medium (25% corn) and low (75% corn) adult food stress (Fig 1). We also can be sure that the 75% corn treatment was benign as our preliminary experiment showed the same growth profile for accessory glands and testes for both less (50%) and more (100%) corn (Additional file 1).

An alternative possibility is that the level of larval stress induces long lasting physiological changes that affect the allocation of resources to reproduction and survival in adults (e.g. [41]). In our study, high quality males that invested in larger eyespan, may thereby have adopted a life history which requires the production of larger accessory glands and testes. In *T. dalmanni*, males with larger eyespan are successful in agonistic interactions with other males [42] and are attractive to females [18-20]. They mate more frequently than males with smaller eyespan during both the dusk and dawn mating periods, and so benefit more from greater investment in their reproductive organs. Larger accessory gland size is strongly associated with higher male mating frequency [13-15] and larger testes presumably allow high mating males to maintain sperm numbers per ejaculate. This may explain why, even in benign conditions of *ad libitum *adult food supply, we still found that males with larger eyespan had larger accessory glands and testes.

In line with this explanation, we found that female fertility was positively correlated with the eyespan of their mating partners. In our assay a single large or small eyespan male was placed with 8 females for 24 hours and we recorded the number of fertile eggs laid by the group of females over the next 30 days, by which time most females would have exhausted their sperm stores [7] (note, we again controlled for body size variation of females in this experiment). This assay gives an indication of the number of sperm successfully transferred when a male has the opportunity for multiple mating. The number of fertile eggs laid increased approximately 8% per millimetre increase in male absolute eyespan. These findings suggest that female preference for large eyespan males potentially could result in higher fertility under natural conditions. In *T. dalmanni*, several females settle on nocturnal aggregations controlled by a single male [12,20,43,44]. The lek holding male mates with the females in his aggregation at dusk and at dawn prior to female dispersal. This corresponds to the conditions in our assay in which fertility was assessed for a single male kept for a limited time with multiple females.

However, we should be cautious about this interpretation. It is unclear what mechanism contributed to the fertility advantage gained by females in our assay. The greater fertility of large eyespan males probably reflected their larger accessory glands which are associated with higher mating frequency, and larger testes which are positively correlated with the number of sperm stored by females following a single copulation [17]. So the group of females housed with large eyespan males probably collectively mated more and received larger amounts of sperm, and as a consequence had higher fertility. However, our experiments do not allow us to distinguish the importance of extra matings, the transfer of more sperm (or other substances) per mating, female effects or other factors contributing to the elevated fertility of large eyespan males. In addition, meiotic drive in *T. dalmanni *is likely to exaggerate differences in fertility in nature and may play an additional selective role with respect to female preference if its occurrence is linked to male eyespan [45] (flies in our experiment lacked any meiotic drive). Further experimentation is required in order to establish the exact mechanism of fertility enhancement by large eyespan males and its relevance to the mating system of *T. dalmanni *in the wild. Our results contrast with a previous study which found no association between male eyespan and the number of fertile eggs laid following a single mating [7]. However, in that study, females were allowed only a single copulation with a large or small eyespan male, whereas multiple mating is typical in this species [46].

Of wider importance, attractive, large eyespan males are likely to suffer from greater ejaculate and sperm depletion due to having more mating opportunities in the dawn and dusk mating periods. Such a loss of male fertility occurs in several other species in which males mate multiply over short periods of time (e.g. [47,48]). Although the reduced fertility of male *T. dalmanni *following mating is known to recover across days [14], this must present an evolutionary dilemma for females as fertility assurance may be higher with an unattractive male rather than with an already mated attractive male. Further understanding of the trade-off between fertility and number of matings is needed to identify the fertility benefits arising from mate choice.

## Conclusion

Our findings are significant in the context of the phenotype-linked fertility hypothesis. This hypothesis states that mate preference for exaggerated male sexual ornaments or displays has evolved to secure fertility benefits for females. Direct tests of this hypothesis are rare, usually incomplete and do not provide unequivocal support [27]. Data obtained in stalk-eyed flies address three fundamental requirements of the hypothesis. First, the ornament or display must be subject to female mate preference. This has been well documented in *T. dalmanni*, where females show strong directional preference for large eyespan males. Second, attractiveness of the male ornament or display must be positively correlated with male reproductive morphology. Here we demonstrated that male eyespan, after controlling for variation in body size, was positively associated with accessory gland length and testis length, over multiple levels of adult nutritional stress. This relationship exists despite male eyespan being fixed prior to the maturation of the reproductive organs. Third, females that mate with males possessing exaggerated ornaments must exhibit higher fertility than females that mate with males with lower trait values. We found that female fertility was positively correlated with the eyespan of their mates under conditions of multiple mating. Together our results support the phenotype-linked fertility hypothesis and indicate that further work on fertility benefits associated with mate preference for male ornaments is warranted.

## Methods

### Male eyespan and reproductive organ size

Experimental flies were taken as eggs from a stock population of *T. dalmanni *which lacks meiotic drive [23]. Groups of 13 eggs were placed in moist cotton-lined Petri dishes. Larvae were reared under high nutritional stress (0.39 g puréed corn per 13 eggs [22]) to generate a large range in both body size and eyespan. Upon eclosion, male thorax length and eyespan were measured as described below.

Male eyespan is highly correlated with body size [23]. In order to maximize variance in eyespan relative to thorax length so as to reveal the individual associations of both traits with internal reproductive organ size, only males with large residual eyespan (25% most positive) and small residual eyespan (25% most negative) were included in the study (using the line of best fit: eyespan = 1.763 (thorax length)^1.6649^). Equal numbers of large and small residual eyespan males were assigned to one of three diets consisting of *ad libitum *amounts of a homogeneous mixture of corn and sucrose (25% sucrose solution containing 3% carboxymethylcellulose, an indigestible starch added to render the viscosity of the sugar solution similar to that of the corn). The three adult diets were 0% corn, 25% corn, 75% corn (% corn by mass) – chosen because they generated the highest levels of variance in reproductive organ size in a pilot study (Additional file 1). Males were housed individually in 500 ml pots and food was changed every two days. Each male was randomly assigned a day for dissection and measurement of their paired accessory glands and testes. A sample of 21 large residual eyespan and 21 small residual eyespan flies per diet was dissected every 14 days for 42 days. These time points were chosen as they represent the periods of most rapid growth, and the final sizes, of both the accessory glands and the testes (Additional file 1).

### Male eyespan and fertility

Adult male flies with large and small residual eyespan were generated under high nutritional stress as described above. Adult males were reared individually in 500 ml plastic pots on an *ad libitum *diet of corn for 34 days to ensure sexual maturity [5]. Each male was then housed with 8 sexually mature females to gain sexual experience (as females will rarely encounter virgin males in the wild). After 14 days, the initial females were discarded and replaced with 8 experimental virgin females. All females used were reared under low nutritional stress (> 2 g puréed corn per 13 eggs [22]) in order to reduce variation in body size and so remove any confounding effects associated with variation in female size. We chose to assess male fertility under conditions of multiple mating, which are typical in this species [43,44], because we had previously shown that there was no fertility difference between females mated once to large or small eyespan males [7].

Males were removed after 24 hours and the eggs laid by each group of 8 experimental females were collected from day 2 to day 32, at 2–3 day intervals. After incubation for four days, eggs were scored as fertile if only the chorion remained or eggs remained intact but exhibited signs of development (e.g. segmental striations). The fecundity (total number of eggs laid) and fertility (total number of fertile eggs) were recorded for each group of 8 females (*N *= 38 large eyespan males and *N *= 37 small eyespan males). Note that there was no effect on female fecundity of male eyespan category (*F*_1,72 _= 0.35, *p *= 0.557) or male thorax length (*F*_1,72 _= 1.21, *p *= 0.275).

### Morphological measurements

Males were ice-anaesthetised and morphological measurements made using a videomicroscope attached to a computer equipped with NIH Image software. Thorax length was measured ventrally from the anterior tip of the prothorax along the midline to the joint between the metathoracic legs and the thorax. Eyespan was defined as the distance between the outer tips of the eyes. Accessory glands and the uncoiled testes were dissected out in phosphate buffered saline on a glass slide and the length of the line that bisected the middle of each organ was recorded [13]. The means of the two accessory gland measurements and the two testis measurements for each fly were used in the analyses. All traits were measured to the nearest 0.01 mm (*N *= 194 large eyespan males and *N *= 191 small eyespan males).

### Statistical analysis

We used general linear models to investigate the effect of eyespan on reproductive organ size. Variance in the length of the accessory glands and the testes (both log-transformed) was analysed in initial models constructed with body size (thorax length), diet, age and all possible interactions. Models were simplified using stepwise elimination of terms that failed to significantly improve the fit of the model (*p *> 0.10). All main effects were treated as continuous variables except diet which was treated as an ordinal categorical variable. After completion of the initial model for each reproductive organ, eyespan was added and the resulting model compared to the initial model for improved fit. Thus, only variance not explained by the initial model could be attributed to eyespan and the effects of eyespan were independent of those of thorax length. Both categorical (large vs. small residual) and absolute values of eyespan were analyzed. Similarly, the interaction between eyespan and diet was added and compared to the previous model (initial model + eyespan) for improved fit. Interactions between eyespan and other variables were not relevant to the study and so were not examined in order to limit the number of steps involved in model building and minimize the risk of type 1 error [49]. From the model, we calculated the regression coefficient of reproductive organ size on absolute eyespan to establish the signalling value of male eyespan (after having dropped thorax length with which absolute eyespan is strongly colinear). We repeated the analysis treating age as a categorical variable and found that this did not alter the qualitative nature of the results reported here.

To investigate the effects of eyespan on fertility, an initial model was constructed including male body size (thorax length) and female fecundity, which were both treated as continuous variables. We then added male eyespan to the initial model to see if it improved the fit. Both categorical (large vs. small residual) and absolute values of eyespan were analyzed. Interactions between eyespan and other variables were not examined to minimize the risk of type 1 error. From the model, we calculated the regression coefficient of female fertility on male absolute eyespan to establish the signalling value of male eyespan. Four males (three small eyespan and one large eyespan) failed to produce any fertile eggs. They were removed from the analysis as they exhibited strong leverage and rendered the distribution of fertility non-normal.

All statistical analyses were conducted using JMP statistical analysis programmes.

## Authors' contributions

DWR participated in the design of the experiments, carried out the experiments, analyzed the data, and wrote the paper. MD helped conduct the experiments. AP participated in the design of the experiments and helped write the paper. TC and KF participated in the design of the experiments and made improvements to the paper.

## Supplementary Material

Additional file 1 Effect of adult nutritional stress on reproductive organ growth. Details of a pilot experiment carried out to identify levels of adult nutritional stress which affected male reproductive organ growth. Click here for file

## References

[B1] Iwasa Y Pomiankowski A Good parent and good genes models of handicap evolution Journal of Theoretical Biology 1999 200 1 97 109 10.1006/jtbi.1999.0979 10479542

[B2] Jennions MD Petrie M Why do females mate multiply? A review of the genetic benefits Biol Rev Camb Philos Soc 2000 75 1 21 64 10.1017/S0006323199005423 10740892

[B3] Arnqvist G Nilsson T The evolution of polyandry: multiple mating and female fitness in insects Animal Behaviour 2000 60 145 164 10.1006/anbe.2000.1446 10973716

[B4] Meier R Baker R A cladistic analysis of Diopsidae (Diptera) based on morphological and DNA sequence data Insect Systematics & Evolution 2002 33 3 325 336

[B5] Baker RH Ashwell RIS Richards TA Fowler K Chapman T Pomiankowski A Effects of multiple mating and male eye span on female reproductive output in the stalk-eyed fly, *Cyrtodiopsis dalmanni* Behavioral Ecology 2001 12 6 732 739 10.1093/beheco/12.6.732

[B6] Kotrba M Sperm transfer by spermatophore in Diptera: New results from the Diopsidae Zoological Journal of the Linnean Society 1996 117 3 305 323 10.1111/j.1096-3642.1996.tb02192.x

[B7] Rogers DW Grant CA Chapman T Pomiankowski A Fowler K The influence of male and female eyespan on fertility in the stalk-eyed fly, *Cyrtodiopsis dalmanni* Animal Behaviour 2006 72 1363 1369 10.1016/j.anbehav.2006.03.027

[B8] Wilkinson GS Amitin EG Johns PM Sex-linked correlated responses in female reproductive traits to selection on male eye span in stalk-eyed flies Integrative and Comparative Biology 2005 45 3 500 510 10.1093/icb/45.3.500 21676795

[B9] Presgraves DC Severance E Wilkinson GS Sex chromosome meiotic drive in stalk-eyed flies Genetics 1997 147 3 1169 1180 9383060 10.1093/genetics/147.3.1169 PMC1208241

[B10] Wilkinson GS Johns PM Kelleher ES Muscedere ML Lorsong A Fitness effects of X chromosome drive in the stalk-eyed fly, *Cyrtodiopsis dalmanni* Journal of Evolutionary Biology 2006 19 1851 1860 10.1111/j.1420-9101.2006.01169.x 17040382

[B11] Wilkinson GS Sanchez MI Sperm development, age and sex chromosome meiotic drive in the stalk-eyed fly, *Cyrtodiopsis whitei* Heredity 2001 87 17 24 10.1046/j.1365-2540.2001.00898.x 11678983

[B12] Cotton S Small J Pomiankowski A Eyespan reflects reproductive quality in wild stalk-eyed flies Evol Ecol 2008 in press

[B13] Baker RH Denniff M Futerman P Fowler K Pomiankowski A Chapman T Accessory gland size influences time to sexual maturity and mating frequency in the stalk-eyed fly, *Cyrtodiopsis dalmanni* Behavioral Ecology 2003 14 5 607 611 10.1093/beheco/arg053

[B14] Rogers DW Chapman T Fowler K Pomiankowski A Mating-induced reduction in accessory reproductive organ size in the stalk-eyed fly *Cyrtodiopsis dalmanni* BMC Evolutionary Biology 2005 5 37 10.1186/1471-2148-5-37 15946387 PMC1180822

[B15] Rogers DW Baker RH Chapman T Denniff M Pomiankowski A Fowler K Direct and correlated responses to artificial selection on male mating frequency in the stalk-eyed fly *Cyrtodiopsis dalmanni* Journal of Evolutionary Biology 2005 18 3 642 650 10.1111/j.1420-9101.2004.00860.x 15842493

[B16] Lorch PD Wilkinson GS Reillo PR Copulation duration and sperm precedence in the stalk-eyed fly *Cyrtodiopsis whitei* (Diptera, Diopsidae) Behavioral Ecology and Sociobiology 1993 32 5 303 311 10.1007/BF00183785

[B17] Fry CL Juvenile hormone mediates a trade-off between primary and secondary sexual traits in stalk-eyed flies Evol Dev 2006 8 2 191 201 10.1111/j.1525-142X.2006.00089.x 16509897

[B18] Hingle A Fowler K Pomiankowski A Size-dependent mate preference in the stalk-eyed fly *Cyrtodiopsis dalmanni* Animal Behaviour 2001 61 589 595 10.1006/anbe.2000.1613 PMC1088732 11410149

[B19] Hingle A Fowler K Pomiankowski A The effect of transient food stress on female mate preference in the stalk-eyed fly *Cyrtodiopsis dalmanni* Proceedings of the Royal Society of London Series B-Biological Sciences 2001 268 1473 1239 1244 10.1098/rspb.2001.1647 PMC1088732 11410149

[B20] Wilkinson GS Reillo PR Female choice response to artificial selection on an exaggerated male trait in a stalk-eyed fly Proceedings of the Royal Society of London Series B-Biological Sciences 1994 255 1342 1 6 10.1098/rspb.1994.0001

[B21] Bjorksten TA Pomiankowski A Fowler K Temperature shock during development fails to increase the fluctuating asymmetry of a sexual trait in stalk-eyed flies Proceedings of the Royal Society of London Series B-Biological Sciences 2001 268 2001/07/17 1475 1503 1510 10.1098/rspb.2001.1575 PMC1088770 11454295

[B22] Cotton S Fowler K Pomiankowski A Condition dependence of sexual ornament size and variation in the stalk-eyed fly *Cyrtodiopsis dalmanni *(Diptera : Diopsidae) Evolution 2004 58 5 1038 1046 15212384 10.1111/j.0014-3820.2004.tb00437.x

[B23] David P Hingle A Greig D Rutherford A Pomiankowski A Fowler K Male sexual ornament size but not asymmetry reflects condition in stalk-eyed flies. Proceedings of the Royal Society of London Series B-Biological Sciences 1998 265 2211 2216 10.1098/rspb.1998.0561

[B24] Buschbeck EK Roosevelt JL Hoy RR Eye stalks or no eye stalks: a structural comparison of pupal development in the stalk-eyed fly *Cyrtodiopsis* and in *Drosophila* Journal of Comparative Neurology 2001 433 4 486 498 10.1002/cne.1155 11304713

[B25] Birkhead TR Fletcher F Male phenotype and ejaculate quality in the zebra finch *Taeniopygia guttata* Proc Biol Sci 1995 262 1365 329 34 10.1098/rspb.1995.0213 8587890

[B26] Sheldon BC Male phenotype, fertility, and the pursuit of extra-pair copulations by female birds Proceedings of the Royal Society of London Series B-Biological Sciences 1994 257 1348 25 30 10.1098/rspb.1994.0089

[B27] Birkhead TR Pizzari T Postcopulatory sexual selection Nature Reviews Genetics 2002 3 2002/04/23 4 262 273 10.1038/nrg774 11967551

[B28] Trivers RL Campbell B Parental investment and sexual selection Sexual Selection and the Descent of Man, 1871-1971 1972 Chicago , Aldine-Atherton 136 179

[B29] Pizzari T Jensen P Cornwallis CK A novel test of the phenotype-linked fertility hypothesis reveals independent components of fertility Proceedings of the Royal Society of London Series B-Biological Sciences 2004 271 51 58 10.1098/rspb.2003.2577 PMC1691554 15002771

[B30] Pizzari T Birkhead TR The sexually-selected sperm hypothesis: sex-biased inheritance and sexual antagonism Biol Rev Camb Philos Soc 2002 77 2 183 209 10.1017/S1464793101005863 12056746

[B31] McGary S Estevez I Bakst MR Pollock DL Phenotypic traits as reliable indicators of fertility in male broiler breeders Poultry Science 2002 81 1 102 111 10.1093/ps/81.1.102 11885889

[B32] Kimball RT Ligon JD MerolaZwartjes M Testicular asymmetry and secondary sexual characters in Red Junglefowl Auk 1997 114 2 221 228

[B33] Wagner WE Harper CJ Female life span and fertility are increased by the ejaculates of preferred males Evolution 2003 57 9 2054 2066 14575327 10.1111/j.0014-3820.2003.tb00385.x

[B34] Wagner WE Harper CJ Females receive a lifespan benefit from male ejaculates in a field cricket. Evolution 2000 55 994 1001 10.1554/0014-3820(2001)055[0994:FRALSB]2.0.CO;2 11430659

[B35] Greenfield MD Rodriguez RL Genotype-environment interaction and the reliability of mating signals Animal Behaviour 2004 68 1461 1468 10.1016/j.anbehav.2004.01.014

[B36] Messina FJ Fry JD Environment-dependent reversal of a life history trade-off in the seed beetle *Callosobruchus maculatus* Journal of Evolutionary Biology 2003 16 3 501 509 10.1046/j.1420-9101.2003.00535.x 14635850

[B37] Johnstone RA Sexual selection, honest advertisement and the handicap principle - reviewing the evidence Biol Rev Camb Philos Soc 1995 70 1 1 65 10.1111/j.1469-185X.1995.tb01439.x 7718697

[B38] Bateman AJ Intra-sexual selection in *Drosophila* Heredity 1948 2 1948/12/01 Pt. 3 349 368 10.1038/hdy.1948.21 18103134

[B39] David P Bjorksten T Fowler K Pomiankowski A Condition-dependent signalling of genetic variation in stalk-eyed flies Nature 2000 406 2000/07/26 6792 186 188 10.1038/35018079 10910358

[B40] Iwasa Y Pomiankowski A Nee S The evolution of costly mate preferences. II. The handicap principle Evolution 1991 45 6 1431 1442 10.2307/2409890 28563835

[B41] Sørensen JG Loeschcke V Larval crowding in *Drosophila melanogaster* induces Hsp70 expression, and leads to increased adult longevity and adult thermal stress resistance Journal of Insect Physiology 2001 47 1301 1307 10.1016/S0022-1910(01)00119-6 12770182

[B42] Panhuis TM Wilkinson GS Exaggerated male eye span influences contest outcome in stalk-eyed flies (Diopsidae) Behavioral Ecology and Sociobiology 1999 46 4 221 227 10.1007/s002650050613

[B43] Burkhardt D de la Motte I Selective pressures, variability and sexual dimorphism in stalk-eyed flies (Diopsidae) Naturwissenschaften 1985 72 221 227 10.1007/BF01195763

[B44] Wilkinson GS Dodson GN Chloe J, Crespi B Function and evolution of antlers and eye stalks in flies The evolution of mating systems in insects and arachnids 1997 Cambridge , Cambridge University Press 310 328

[B45] Wilkinson GS Presgraves DC Crymes L Male eye span in stalk-eyed flies indicates genetic quality by meiotic drive suppression Nature 1998 391 276 279 10.1038/34640

[B46] Reguera P Pomiankowski A Fowler K Chapman T Low cost of reproduction in female stalk-eyed flies, *Cyrtodiopsis dalmanni* Journal of Insect Physiology 2004 50 1 103 108 10.1016/j.jinsphys.2003.10.004 15037098

[B47] Thwaites C The comparative effects of undernutrition, exercise and frequency of ejaculation on the size and tone of the testes and on semen quality in the ram Anim Reprod Sci 1995 37 299 309 10.1016/0378-4320(94)01343-K

[B48] Preston BT Stevenson IR Pemberton JM Wilson K Dominant rams lose out by sperm depletion Nature 2001 409 6821 681 682 10.1038/35055617 11217847

[B49] Foster DP Stine RA Honest confidence intervals for the error variance in stepwise regression Journal of Economic and Social Measurement 2006 31 89 102

