# Supplementary material for: Male sexual ornament size is positively associated with reproductive morphology and enhanced fertility in the stalk-eyed fly Teleopsis dalmanni
Source: BMC Evol Biol. 2008 Aug 18;8:236. doi: 10.1186/1471-2148-8-236 (PMC2562384; doi:10.1186/1471-2148-8-236)
Supplement: Additional file 1 — Effect of adult nutritional stress on reproductive organ growth. Details of a pilot experiment carried out to identify levels of adult nutritional stress which affected male reproductive organ growth. [file 1471-2148-8-236-S1.pdf]

Additional File 1

Effect of adult nutritional stress on reproductive organ growth

A pilot experiment was carried out to identify levels of adult nutritional stress which affected male reproductive organ growth. Males were produced as described in the Methods except that larvae were reared under low nutritional stress (>2g of puréed corn per 13 eggs) to minimize variation in body size and eyespan and so minimize reproductive organ allometry. To further reduce the effects of allometric scaling, body size was measured at eclosion and males with body length outside the range of one standard deviation above and below the mean were discarded. Remaining males were assigned to one of five adult diets: 0% corn, 25% corn, 50% corn, 75% corn, 100% corn. Reproductive organs were measured every 7 days for 49 days, with a total of 11 flies per diet dissected on each day. Data analysis was performed using a general linear model including body size, diet and age as predictors (both diet and age were treated as categorical variables, allowing comparison of differences between levels of each factor).

We were able to eliminate the positive allometry of both reproductive organs. Body size failed to predict either accessory gland length ($F_{1,371} = 0.23$, $p = 0.6307$) or testis length ($F_{1,369} = 0.01$, $p = 0.9346$). Age was a significant predictor of reproductive organ size; both accessory gland length ($F_{6,371} = 316.70$, $p < 0.0001$) and testis length ($F_{6,369} = 16.91$, $p < 0.0001$) increased with age. Multiple pairwise comparisons (Tukey HSD) revealed significant increases in accessory gland length over each of the first 4 weeks (day 8 < 15 < 22 < 36) but no significant difference between the last three weeks. Similarly, testis length increased over each of the first 2 weeks (day 8 < 15) and between day 15 and days 36-50. There was no

significant difference in testis length between the last three weeks.

Diet also had important effects on reproductive organ size; both accessory gland length ($F_{4, 371}$ = 89.72, $p < 0.0001$) and testis length ($F_{4,369}$ = 5.97, $p = 0.0001$) decreased with increasing levels of nutritional stress. Multiple pairwise comparisons (Tukey HSD) revealed significant differences in accessory gland length between 0% corn, 25% corn, and the three least stressful diets (50% corn, 75% corn, 100%) which were not significantly different from one another. Similarly, testis length was significantly smaller in males raised on 0% corn than in the three least stressful diets (no significant difference was observed between the 25% corn group and any other diet).

As the three least stressful diets were equivalent, we chose to examine the effects of eyespan on reproductive organ growth across three different levels of adult nutritional stress: 0%, 25%, and 75% corn. We also chose to measure reproductive organs at three time points: 14 days, 28 days and 42 days post-eclosion to include periods of rapid growth and the attainment of final size for both the testes and the accessory glands.

Figure S1

The effect of adult nutritional stress on growth of (a) the accessory glands and (b) the testes, in male stalk-eyed flies raised under low larval stress. Organ growth trajectories for flies exposed to one of five different levels of nutritional stress. Data points represent the least squares means ± s.e. log reproductive organ length.

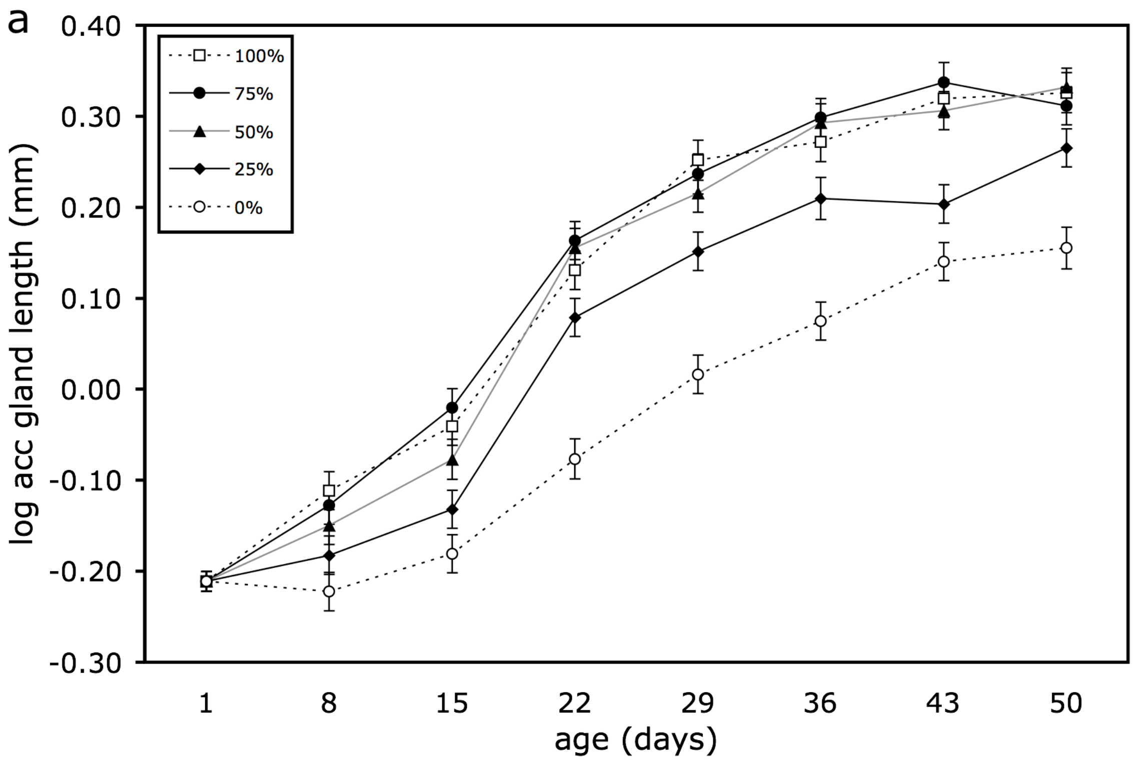
a
log acc gland length (mm)
0.40
0.30
0.20
0.10
0.00
-0.10
-0.20
-0.30
100%
75%
50%
25%
0%
1
8
15
22
29
36
43
50
age (days)

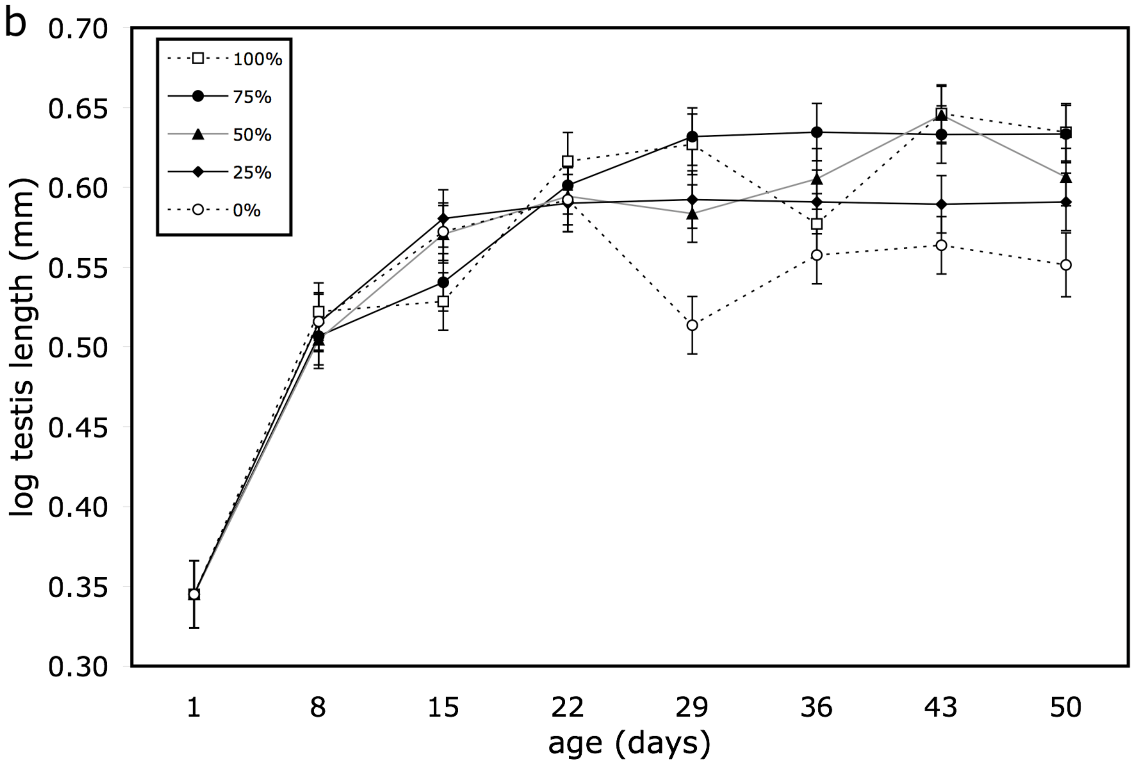
b
log testis length (mm)
0.70
0.65
0.60
0.55
0.50
0.45
0.40
0.35
0.30
100%
75%
50%
25%
0%
1
8
15
22
29
36
43
50
age (days)
